# Structural Consideration in Designing Organotin Polyethers to Arrest the Growth of Breast Cancer Cells *In Vitro*

**DOI:** 10.3390/ma4040801

**Published:** 2011-04-15

**Authors:** Charles E. Carraher, Michael R. Roner, Kimberly Shahi, Girish Barot

**Affiliations:** 1 Department of Chemistry and Biochemistry, Florida Atlantic University, Boca Raton, FL 33431, USA; E-Mail: carraher@fau.edu (C.E.C.); 2 Florida Center for Environmental Studies, Palm Beach Gardens, FL 33410, USA; 3 Department of Biology, University of Texas Arlington, Arlington, TX 76019, USA; 4 Department of Biological Sciences, University of North Texas, Denton, TX 76203, USA; E-Mail: kimberly.shahi@unt.edu (K.S.); 5 Department of Biology, Boston University, Boston, MA 02215, USA; E-Mail: girishbarod@yahoo.com (G.B.)

**Keywords:** tin-containing polymers, cancer, breast cancer, organotin compounds, organotin polyethers, estrogen receptor positive breast cancer, estrogen-independent breast cancer

## Abstract

The ability to inhibit cancer is inherent in organotin materials yet the structural relationships that regulate/direct this activity remains unknown. We measured antitumor activity using a matched pair of cell lines MDA-MB-231 cells that are estrogen-independent, estrogen receptor negative and MCF-7 cells, a cell line that is estrogen receptor (ER) positive. Those polyethers that contained a O-phenyl unit were able to significantly inhibit the non-estrogen sensitive cell line but were much less effective against the estrogen sensitive cell line; that is, the human breast cancer cell line MDA-MB-231 showed better test results for polymers derived from diols containing the O-phenyl moiety than the breast cancer cell line MCF-7, a well-characterized estrogen receptor positive control cell line. Those polyethers that did not contain the O-phenyl unit inhibited both cell lines approximately the same. The differential activity of the O-phenyl-containing polyethers is likely due to the estrogen-sensitive cells combining with some of the organotin polyethers minimizing their ability to inhibit cell growth.

## 1. Introduction

The ability to inhibit cancer by organotin materials has been known for about 80 years [1]. Even so, identification of structural relationships has not been accomplished [2,3,4,5,6,7]. We have been involved in the synthesis and structural and biological characterization of metal-containing polymers. Recently, much of the biological effort has focused on organotin, platinum and Group IVB metallocene-containing polymers [3,5,8,9,10,11,12,13,14,15,16,17,18,19,20,21,22,23,24,25,26]. The topics of organotin polymers [3,5], platinum polymers [22] and Group IVB metallocene-containing polymers [24] have been recently reviewed. The interaction of monomeric or small molecule organotin compounds and breast cancer has been previously described [27,28,29,30,31,32].

We have noticed in our general testing of the ability of various organotin polyethers to inhibit different cancer cell lines that in certain cases the human breast cancer cell line MDA-MB-231, that is estrogen-independent, estrogen receptor negative, showed better test results than the breast cancer cell line MCF-7, a well-characterized estrogen receptor positive control cell line (cells are positive for cytoplasmic estrogen receptors) and therefore are a useful *in vitro* model of breast cancer to study the role of estrogen in breast cancer. Here, we describe some of these results.

## 2. Results and Discussion

### 2.1. Measurement Types

Two types of measurements are generally employed to report the ability of materials to inhibit cell growth. The first is the growth inhibition, GI, value. Generally the concentration at which 50% growth inhibition occurs, GI_50_, is reported. The second value is the chemotherapeutic index (CI) value. The CI is employed to compare the toxicity of a drug on normal cell lines (or other “base-line” cell line) to its toxicity to a cancer (or second) cell line. The CI_50_ is the GI_50_ drug concentration for the normal (or base line) cell line divided by the GI_50_ drug concentration for the cancer cell line. Values in excess of two are generally considered significant.

To gain a better picture of the relationship between polymer and possible preference for one cell line over the other one additional column have been added to each table. For the GI_50_ values, a column noting the ratio of the GI_50_ for the MDA cell line divided by the GI_50_ for the MCF-7 cell line, MDA/MCF-7, has been added. Here, values greater than one are consistent with lower concentrations of drug inhibiting the growth of the MCF-7 cells compared to the MDA cells. Values less than one are consistent with inhibition of the MDA cells occurring at lower concentrations than for the MCF-7 cells. When describing the CI some researchers employ the similar terms ED or effective dose in place of the GI value for the cancer cells and LD or lethal dose in place of GI for the healthy cell line, here the WI-38 cell line (strain line ATCC CCL-75). Thus, CI_50_ = LD_50_/ED_50_. For the CI_50_ values the added column contains the CI_50_ for the MDA cell line divided by the CI_50_ for the MCF-7 cell line, CI-MDA/CI-MCF-7. Here, values greater than one are consistent with inhibition of the MDA cells occurring at lower concentrations than for the MCF-7 cells. And, values less than one are consistent with lower concentrations of drug inhibiting the growth of the MCF-7 cells compared to the MDA cells. The values contained in the added columns should be related since the CI values are “normalized” with the same WI-38 cell line values.

### 2.2. Aliphatic-Derived Organotin Polyethers

**Figure 1:**
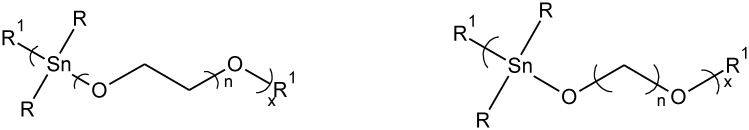
Repeat unit for the polymers derived from the reaction of organotin dihalides with various ethylene glycols (left) and with methylene oxide diols (right) where R is butyl and R^1^ represents simple chain extension.

Table 1 and Table 2 contain data from organotin polyethers derived from aliphatic diols (Figure 1).

**Table 1:** GI_50_ concentrations (micrograms/mL) for organotin polyethers from ethylene glycols and methylene diols for MDA and MCF-7 cell lines.

Sample	Cell Line			
	WI-38	MDA	MCF-7	MDA/MCF-7
Bu_2_Sn/Ethylene glycol	0.90(.10)	0.30(.023)	0.60(.05)	0.50
Bu_2_Sn/Diethylene glycol	1.20(.10)	1.20(.10)	1.20(.10)	1.0
Bu_2_Sn/Triethylene glycol	1.10(.10)	1.20(.10)	1.20(.11)	1.0
Bu_2_Sn/Pentaethylene glycol	0.05(.01)	0.90(.01)	1.20(.01)	0.75
Bu_2_Sn/PEG(400),DMSO	3.50(.29)	1.90(.16)	2.80(.22)	0.68
Bu_2_Sn/PEG(400),H_2_O	0.28(.03)	2.40(.22)	1.40(.10)	1.7
Bu_2_Sn/PEG(8000),DMSO	0.11(.01)	3.20(.29)	3.20(.30)	1.0
Bu_2_Sn/PEG(8000),H_2_O	1.00(.10)	10.00(.93)	10.00(.96)	1.0
Bu_2_Sn/PEG(10000),DMSO	4.20(.33)	10.00(.89)	5.80(.47)	1.7
Bu_2_Sn/PEG(10000),H_2_O	1.00(.10)	10.00(.97)	10.00(1.0)	1.0
Bu_2_Sn/Ethylene glycol	0.90(.10)	0.30(.02)	0.60(.05)	0.50
Bu_2_Sn/1,3-Propanediol	0.05(.01)	0.90(.10)	1.1(0.10)	0.82
Bu_2_Sn/1,4-Butanediol	0.06(.01)	0.22(.02)	0.15(.02)	1.5
Bu_2_Sn/1,5-Pentanediol	0.05(.01)	0.09(.04)	0.20(.01)	0.45
Bu_2_Sn/1,6-Hexanediol	0.05(.01)	0.35(.04)	0.22(.02)	1.6
Bu_2_Sn/1,7-Heptanediol	0.04(.01)	0.10(.01)	0.20(.01)	0.50
Bu_2_Sn/1,8-Octanediol	0.02(.01)	0.09(.01)	0.22(.03)	0.41

Values given in ( ) are standard deviations for each set of measurements.

**Table 2:** Chemotherapeutic Index-50% for the organotin polyethers from ethylene glycols and methylene diols for MDA and MCF-7 cell lines.

Sample	Cell Line			
	WI-38/	WI-38/	WI-38/	CI-MDA/
	WI-38	MDA	MCF-7	CI-MCF-7
Bu_2_Sn/Ethylene glycol	1.0	3.0	1.5	2.0
Bu_2_Sn/Diethylene glycol	1.0	1.0	1.0	1.0
Bu_2_Sn/Triethylene glycol	1.0	0.92	0.92	1.0
Bu_2_Sn/Pentaethylene glycol	1.0	0.06	0.04	1.5
Bu_2_Sn/PEG(400),DMSO	1.0	1.8	1.3	1.4
Bu_2_Sn/PEG(400),H_2_O	1.0	0.12	0.20	0.60
Bu_2_Sn/PEG(8000),DMSO	1.0	0.03	0.03	1.0
Bu_2_Sn/PEG(8000),H_2_O	1.0	0.10	0.10	1.0
Bu_2_Sn/PEG(10000),DMSO	1.0	0.42	0.72	0.58
Bu_2_Sn/PEG(10000),H_2_O	1.0	0.10	0.10	1.0
Bu_2_Sn/Ethylene glycol	1.0	21	5.2	4.0
Bu_2_Sn/1,3-Propanediol	1.0	0.43	0.17	2.5
Bu_2_Sn/1,4-Butanediol	1.0	1.6	1.1	1.5
Bu_2_Sn/1,5-Pentanediol	1.0	4.0	0.86	4.7
Bu_2_Sn/1,6-Hexanediol	1.0	1.0	0.79	1.3
Bu_2_Sn/1,7-Heptanediol	1.0	3.6	0.86	4.2
Bu_2_Sn/1,8-Octanediol	1.0	1.6	0.31	5.2

Characterization of these materials has been described [11,33]. The values of GI_50_ MDA/ GI_50_ MCF-7 as well as the corresponding CI_50_ MDA/ CI_50_ MCF-7 values are around one consistent with the organotin polyethers not having a marked preference for either cell line. The aliphatic diols themselves do not exhibit cancer cell inhibition. The values of GI_50_ MDA/ GI_50_ MCF-7 are about one (average = 0.95) and are consistent with the organotin polyethers not having a preference for either cell line. The corresponding CI_50_ MDA/ CI_50_ MCF-7 values are around two (average = 2.0) consistent with the organotin polyethers having some preference for inhibiting the non-estrogen MDA cell line at lower concentration of drug than the estrogen-sensitive MCF-7 cells.

### 2.3. Hydroquinone and Hydroquinone-Derived Organotin Polyethers

**Figure 2:**
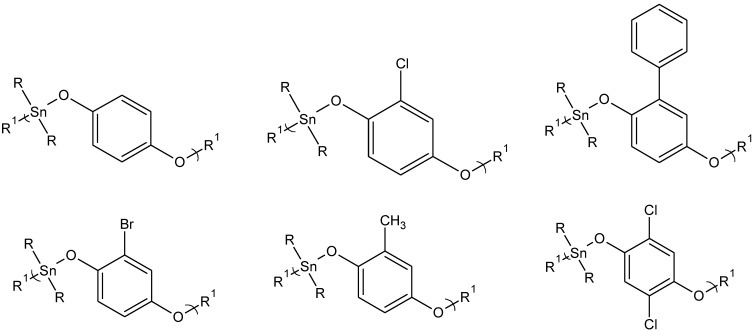
Representative structures for some of the hydroquinone and hydroquinone-derived polyethers. The diols are from left to right, top: hydroquinone itself, chlorohydroquinone, phenylhydroquinone, and left to right, bottom: bromohydroquinone, methylhydroquinone, and 2,5-dichlorohydroquinone where R is butyl and R^1^ represents simple chain extension.

Characterization of these products has been described [34]. Table 3 and Table 4 contain similar GI_50_ and CI_50_ data for organotin polyethers derived from hydroquinone and hydroquinone derivatives (Figure 2).

The GI_50_ MDA/ GI_50_ MCF-7 values are much less than one (average = 0.068) while the CI_50_ MDA/ CI_50_ MCF-7 values are much greater than one (average = 20). These are markedly different than for the organotin polyethers derived from aliphatic diols. Here, inhibition occurs at a much lower concentration for the MDA cell line in comparison to the MCF-7 cell line. The diols in this study showed little or no inhibition of the cancer cell lines.

**Table 3:** GI_50_ concentrations (micrograms/mL) for dibutyltin polyethers from hydroquinone and hydroquinone derivatives for MDA and MCF-7 cell lines.

Sample	Cell Line			
	WI-38	MDA	MCF-7	MDA/MCF-7
Methoxyhydroquinone	2.0(0.3)	0.09(0.01)	1.7(0.4)	0.053
Tert-Butylhydroquinone	1.8(0.5)	0.23(0.01)	2.4(0.5)	0.096
2,5-Di-tert-Butylhydroquinone	2.2(0.5)	0.22(0.01)	1.8(0.5)	0.12
Methylhydroquinone	2.6(0.5)	0.036(0.1)	1.7(0.4)	0.021
Phenylhydroquinone	0.21(.03)	0.11(0.01)	1.7(0.4)	0.065
Hydroquinone	2.0(0.5)	0.045(0.01)	1.7(0.5)	0.026
2,3-Dicyanohydroquinone	2.4(0.5)	0.22(0.09)	1.9(0.5)	0.12
Bromphydroquinone	0.25(0.1)	0.085(0.01)	2.7(0.4)	0.031
Chlorohydroquinone	1.8(0.3)	0.086(0.01)	1.7(0.4)	0.051
2,5-Dichlorohydroquinone	1.9(0.3)	0.38(0.09)	2.6(0.5)	0.15
Tetrachlorohydroquinone	2.2(0.5)	0.12(0.01)	3.9(0.5)	0.031
2,5-Dihydroxybenzaldehyde	2.0(0.5)	0.13(0.01)	2.4(0.5)	0.054

Values given in ( ) are standard deviations for each set of measurements.

**Table 4:** Chemotherapeutic Index-50% for the polymers formed from reaction of dibutyltin dichloride and hydroquinone and hydroquinone derivatives for MDA and MCF-7 cell lines.

Sample	Cell Line			
	WI-38/	WI-38/	WI-38/	CI-MDA/
	WI-38	MDA	MCF-7	CI-MCF-7
Methoxyhydroquinone	1.0	21	1.2	1.8
Tert-Butylhydroquinone	1.0	7.6	0.72	11
2,5-Di-tert-Butylhydroquinone	1.0	9.8	1.2	8.2
Methylhydroquinone	1.0	71	1.5	47
Phenylhydroquinone	1.0	1.9	0.12	16
Hydroquinone	1.0	43	1.1	39
2,3-Dicyanohydroquinone	1.0	11	1.2	9.2
Bromphydroquinone	1.0	2.9	0.090	32
Chlorohydroquinone	1.0	21	1.1	19
2,5-Dichlorohydroquinone	1.0	4.9	0.72	6.8
Tetrachlorohydroquinone	1.0	19	0.57	33
2,5-Dihydroxybenzaldehyde	1.0	15	0.84	18

### 2.4. Hormone-Derived Organotin Polyethers

In our study of a variety of organotin polyethers we included the study of two related hormone-containing products. Characterization of these products has been reported [10,35,36].

The first is diethylstilbestrol (DES). Diethylstilbestrol (4,4’[(1E)-1,2-ethenediyl]bisphenol), is a synthetic estrogen that mimics estrogen, one of the primary ovarian hormones. It is known by a number of common names including stilbesterol and stilboestrol and sold under a number of names including Apstil, cyren A, distilbene, and stilbetin.

DES was first used in 1938 for women in an effort to prevent miscarriage or premature deliveries. In 1953, a double-blind study showed that DES did little to improve premature deliveries or miscarriage. Even so, it was still widely marketed until the early 1970s for this use. By 1971 it was estimated that 5 to 10 million people were exposed to DES. In 1971 the Food and Drug Administration issued a Drug Bulletin advising physicians to halt prescribing DES. DES was linked to a rare vaginal cancer in female offspring. Further research has shown that DES is a teratogen that can cause malformation of an embryo or fetus.

DES is currently used with animals. Its primary use is to treat urinary incontinence in spayed female cats and dogs. It has also been used to prevent unwanted pregnancy in dogs and cats. DES has been used to treat breast and prostate cancer but its use is limited because of relatively poor water solubility and a wide range of dose-related toxicities that includes nausea and vomiting, venous and arterial thrombosis, and fluid retention. The use of estrogens as potent antiandrogens in hormonal therapy of metastatic prostate cancer has also been described. Thus, there exist several studies that indicate the potential usefulness of DES as a positive drug in the treatment of specific cancers.

The second hormone was dienestrol. Dienestrol,(4-[4-(hydroxyphenyl)hexa-2,4-dien-3-yl]phenol, is one of the most widely used sex hormones. It was initially synthesized by Dodd and others in 1938 and initially patented by both Boots and Hoffman-La Roche in 1949. In the popular literature it is often confused with DES, diethylstilbestrol, but it is a distinct hormone with its own chemical and biological properties. It is sold under a variety of trade names including Farmacyrol, Lipamone, and Retalon-Oral.

Dienestrol is widely used in hormone therapy, mainly hormone replace therapy or more precisely, estrogen replacement therapy.

**Figure 3:**
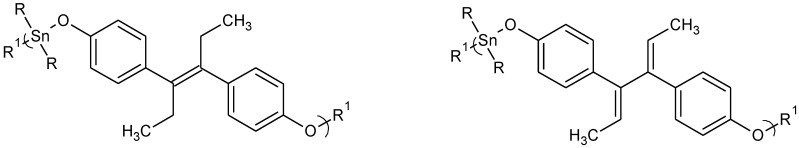
Repeat unit for organotin polymers from the reaction of organotin dihalides with diethylstilbestrol (DES), left, and with dienestrol, right.

Table 5 and Table 6 contain data for organotin polyethers derived from the hormones diethylstilbestrol, DES, and dienestrol (Figure 3).

**Table 5:** GI_50_ concentrations (micrograms/mL) for organotin polyethers derived from diethylstilbestrol (DES) and dienestrol for MDA and MCF-7 cell lines.

Sample	Cell Line			
	WI-38	MDA	MCF-7	MDA/MCF-7
DES	0.25(0.2)	0.05(0.01)	0.64(0.05)	0.078
Me_2_Sn/DES	1.60(0.5)	0.47(0.04)	0.66(0.05)	0.71
Et_2_Sn/DES	0.05(0.01)	0.16(0.01)	0.55(0.05)	0.29
Pr_2_Sn/DES	2.30(0.5)	0.09(0.01)	0.66(0.05)	0.14
Bu_2_Sn/DES	2.50(0.5)	0.05(0.01)	0.62(0.05)	0.081
Cy_2_Sn/DES	0.22(0.02)	0.21((0.02)	0.50(0.05)	0.42
Ph_2_Sn/DES	2.30(0.5)	0.11(0.02)	0.65(0.05)	0.17
Dienestrol	0.25(0.2)	0.11(.02)	0.44(0.05)	0.25
Me_2_Sn/Dienestrol	1.5(.5)	0.13(.06)	0.76(0.06)	0.17
Et_2_Sn/Dienestrol	1.4(.5)	0.04(.01)	0.81(0.06)	0.049
Pr_2_Sn/Dienestrol	0.31(.2)	0.21(.02)	0.69(0.05)	0.30
Bu_2_Sn/Dienestrol	0.06(.01)	0.03(.01)	0.76(0.05)	0.039
Cy_2_Sn/Dienestrol	0.26(.2)	0.24(.02)	0.70(0.05)	0.34
Ph_2_Sn/Dienestrol	0.19(.2)	0.31(.04)	0.70(0.05)	0.44

Values given in ( ) are standard deviations for each set of measurements.

**Table 6:** Chemotherapeutic Index-50% for the organotin polyethers derived from diethylstilbestrol (DES) and dienestrol for MDA and MCF-7 cell lines.

Sample	Cell Line			
	WI-38/	WI-38/	WI-38/	CI-MDA/
	WI-38	MDA	MCF-7	CI-MCF-7
DES	1.0	5.0	0.39	13
Me_2_Sn/DES	1.0	3.4	2.5	1.4
Et_2_Sn/DES	1.0	0.31	0.09	3.4
Pr_2_Sn/DES	1.0	2.6	3.5	0.74
Bu_2_Sn/DES	1.0	50	4.0	13
Cy_2_Sn/DES	1.0	1.0	4.4	0.23
Ph_2_Sn/DES	1.0	21	3.5	6.0
Dienestrol	1.0	2.2	0.6	3.7
Me_2_Sn/Dienestrol	1.0	12	2	6.0
Et_2_Sn/Dienestrol	1.0	35	1.7	20
Pr_2_Sn/Dienestrol	1.0	1.6	0.5	3.2
Bu_2_Sn/Dienestrol	1.0	2	0.1	20
Cy_2_Sn/Dienestrol	1.0	1.1	0.4	2.8
Ph_2_Sn/Dienestrol	1.0	0.6	0.3	2.0

Here, the GI_50_ MDA/ GI_50_ MCF-7 are generally much smaller than one (average = 0.30 for DES and 0.22 for dienestrol) while the CI_50_ MDA/ CI_50_ MCF-7 values are generally much larger than one (average = 4.0 for DES and 8.5 for dienestrol). The values are consistent with these organotin polyethers preferentially inhibiting the MDA non-estrogen sensitive cancer cell line. Further, the values for DES and dienestrol themselves are consistent with a preferential inhibition of the MDA non-estrogen cancer cell line. DES is effective against estrogen receptor positive (ER+) tumors such as the MCF-7 cell line [37,38,39,40,41,42,43,44,45,46]. It is possible that some of the drug is bound to the estrogen receptors in the MCF-7 cells making the drug unavailable to act within the cell.

Thus, the results for the two hormones are similar to those found for the hydroquinone and hydroquinone-derived products described in Table 3 and Table 4 but different from those derived from simple aliphatic diols Table 1 and Table 2. DES and dienestrol have a structural similarity to the hydroquinone products in that all possess an O-phenylene linkage to the organotin. The organotin polyethers derived from aliphatic diols do not possess this linkage.

### 2.5. Organotin Monomers

Table 7 and Table 8 contain GI_50_ and CI_50_ results for the organotin monomers. The GI_50_ MDA/ GI_50_ MCF-7 values are about one (average = 1.07) as are the CI_50_ MDA/ CI_50_ MCF-7 values (average = 1.06). Thus, the results are similar to those found for the aliphatic-derived diols given in Table 1 and Table 2 with no marked preference for either cell line. It appears then that the difference in preference between the two cell lines is due to what is linked to the orgnaotin and not the organotin itself.

**Table 7:** GI_50_ concentrations (micrograms/mL) for organotin monomers for MDA and MCF-7 cell lines.

Sample	Cell Line			
	WI-38	MDA	MCF-7	MDA/MCF-7
Me_2_SnCl_2_	0.22(.1)	0.44(.1)	0.66(0.1)	0.67
Et_2_SnCl_2_	0.20(.1)	0.64(.1)	0.77(0.1)	0.83
Pr_2_SnCl_2_	0.25(.1)	0.47(.1)	0.45(0.1)	1.0
Bu_2_SnCl_2_	0.20(.05)	1.40(.12)	0.70(0.06)	2.0
Ph_2_SnCl_2_	0.25(.1)	0.76(.1)	0.68(0.1)	1.1
Cy_2_SnCl_2_	0.20(.1)	0.45(.1)	0.59(0.1)	0.76
Oc_2_SnCl_2_	0.30(.1)	0.65(.1)	0.70(0.1)	0.93
Bz_2_SnCl_2_	0.20(.1)	0.75(.1)	0.60(0.10)	1.3

Values given in ( ) are standard deviations for each set of measurements.

**Table 8:** Chemotherapeutic Index-50% for organotin monomers for MDA and MCF-7 cell lines.

Sample	Cell Line			
	WI-38/	WI-38/	WI-38/	CI-MDA/
	WI-38	MDA	MCF-7	CI-MCF-7
Me_2_SnCl_2_	1.0	0.50	0.33	1.5
Et_2_SnCl_2_	1.0	0.31	0.26	1.2
Pr_2_SnCl_2_	1.0	0.63	0.56	1.1
Bu_2_SnCl_2_	1.0	0.17	0.29	0.59
Ph_2_SnCl_2_	1.0	0.33	0.37	0.89
Cy_2_SnCl_2_	1.0	0.44	0.34	1.3
Oc_2_SnCl_2_	1.0	0.46	0.43	1.1
Bz_2_SnCl_2_	1.0	0.27	0.33	0.82

## 3. Experimental Section

### 3.1. Synthesis and Physical Characterization

Reactions were carried out using the interfacial polycondensation technique. Briefly, an aqueous solution (30 mL) containing the diol (0.00300 mol) and sodium hydroxide (0.0060 mol) was transferred to a one quart Kimax emulsifying jar fitted on top of a Waring Blender (model 1120; no load speed of about 18,000 rpm; reactions were carried out at about 25 °C). Stirring was begun and a hexane solution (30 mL) containing the diorganotin dichloride (0.00300 mol) was rapidly added (about 3-4 seconds) through a hole in the jar lid using a powder funnel. The resulting solution was blended for 15 seconds. The precipitate was recovered using vacuum filtration and washed several times with deionized water and hexane to remove unreacted materials and unwanted by-products. The solid was washed onto a glass petri dish and allowed to dry at room temperature. Chain length was determined employing light scattering photometry using a Brice-Phoenix BP 3000 Universal Light Scattering Photometer. Refractive indices were obtained using a Bauch & Lomb Model 3-L refractometer. Additional analysis was carried out including Mössbauer spectroscopy, infrared spectroscopy, NMR spectroscopy, and MALDI MS [4,10,11,13,18,33,34,35,36].

### 3.2. Biological Characterization

The cell lines MDA-MB-231, that is estrogen-independent, estrogen receptor negative, and the breast cancer cell line MCF-7 a cell line isolated in 1970 from a 69-year-old Caucasian woman. MCF-7 is the acronym of Michigan Cancer Foundation. MCF-7 cells are a well-characterized estrogen receptor (ER) positive control cell line (cells are positive for cytoplasmic estrogen receptors) and therefore are a useful *in vitro* model of breast cancer to study the role of estrogen in breast cancer. Cells are also positive for cytokeratin and negative for desmin, endothelin, GFAP, neurofilament, vimentin. Both cell lines were obtained from NCI and maintained in MEM supplemented with 10% fetal bovine serum at 37 °C in a 5% carbon dioxide atmosphere.

For testing of the compounds, cells were harvested, counted, and plated into 96-well plates at 1 × 10^4^ cells per well in MEM supplemented with 10% fetal bovine serum, and incubated for 24 hours at 37 °C in a 5% carbon dioxide atmosphere. A stock solution of the compound was prepared in DMSO at a known concentration. On day two the cell media was removed and replaced with RPMI-1640 supplemented with 10% fetal bovine serum and the indicated drug concentrations. Seventy-two hours later the cells were assayed for proliferation using the CellTiter 96^®^ Aqueous One Solution Cell Proliferation Assay by Promega Corporation. Assays are performed by adding a small amount of the CellTiter 96 Aqueous One Solution Reagent directly to culture wells, incubating for 1–4 hours and then recording absorbance at 490 nm with a 96-well plate reader. The quantity of formazan product as measured by the amount of 490 nm absorbance is directly proportional to the number of living cells in culture.

All cytotoxicity values are calculated against a base-line value for each line that was generated from “mock-treatment” of the normal and tumor cells lines with media supplemented with all diluents used to prepare the chemotherapeutic compounds. For example, if the compounds were dissolved in DMSO and serial dilutions prepared in MEM to treat the cells, then the mock-treated cells were “treated” with the same serial dilutions of DMSO without added chemotherapeutic compound. This was done to ensure that any cytotoxicity observed was due to the activity of the compound and not the diluents. For the studies reported here, the mock-treatment never resulted in a loss of cell viability of more than one percent, demonstrating that the activity observed was not due to cytotoxicity of any of the diluents used, but was due to activity of the tested compounds.

## 4. Conclusions

The results are consistent with the organotin polyethers derived from diols containing a O-phenyl moiety exhibiting a much lowered ability to inhibit the MCF-7 estrogen-sensitive cell line in comparison to the MDA non-estrogen cell line. By comparison, those organotin polyethers derived from simple aliphatic diols that do not contain an O-phenyl linkage to the tin exhibit little or no preference towards inhibition of either cell line.

Recently, the ability of ionic tin to exert estrogen-like activity in MCF-7 breast cancer cells was reported [37]. In this paper [37], ionic tin, along with a number of other metal ions, activated the estrogen receptor-α (ERα). Thus, ionic tin has been shown to interact with MCF-7 cancer cells. Our study employs covalently-bonded tin and it is not known if the current interaction with the two breast cell lines is related to the results of this study, but it is believed that the presence of the organotin moiety alone is not sufficient to itself be responsible for this difference based on the lack of differentiation between the two cell lines found for the organotin monomers themselves. A better conclusion is that the presence of tin allows for all of the organotin polymers to exhibit some inhibition of the cancer cell lines. If the biological activity involves interaction with vacant d-orbitals on the tin, then there may be some similarity since the organotin moiety in the present polyethers also has available d-orbitals. The ER is a known metal-binding protein and this binding probably involves vacant d-orbitals on the metal ions. It is possible that both breast cancer cell lines interact with the available d-orbitals on the tin and that the differentiation is a result of more than simple interaction with the d-orbitals on the tin. Since organotin polyethers containing the Sn-O-phenyl grouping show a lowered ability to inhibit the MCF-7 cell lines then this grouping may facilitate this linking between the organotin polyether and some site associated with the cancer cell line removing the organotin from exerting its inhibitory action at some other site within the MCF-7 cell.

**Figure 4:**
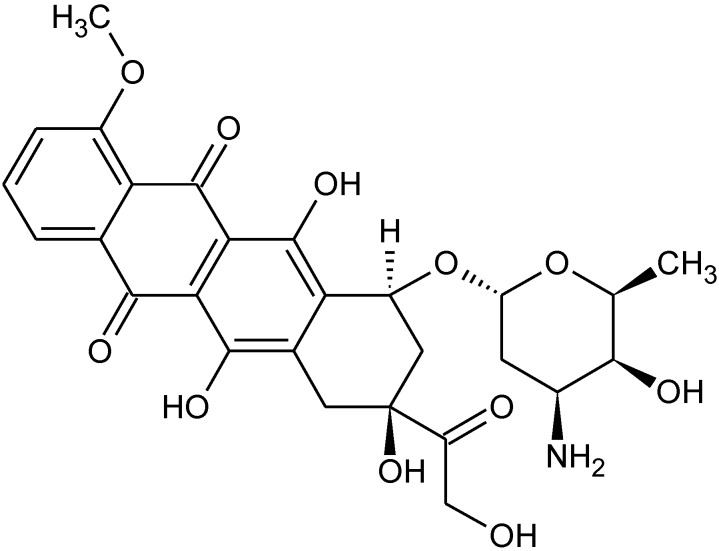
Doxorubicin.

**Figure 5:**
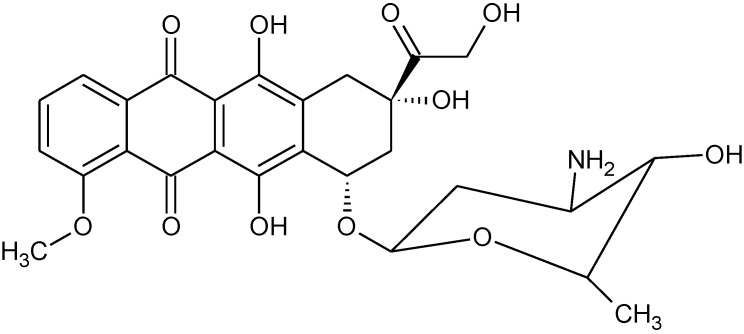
Epirubicin.

**Figure 6:**
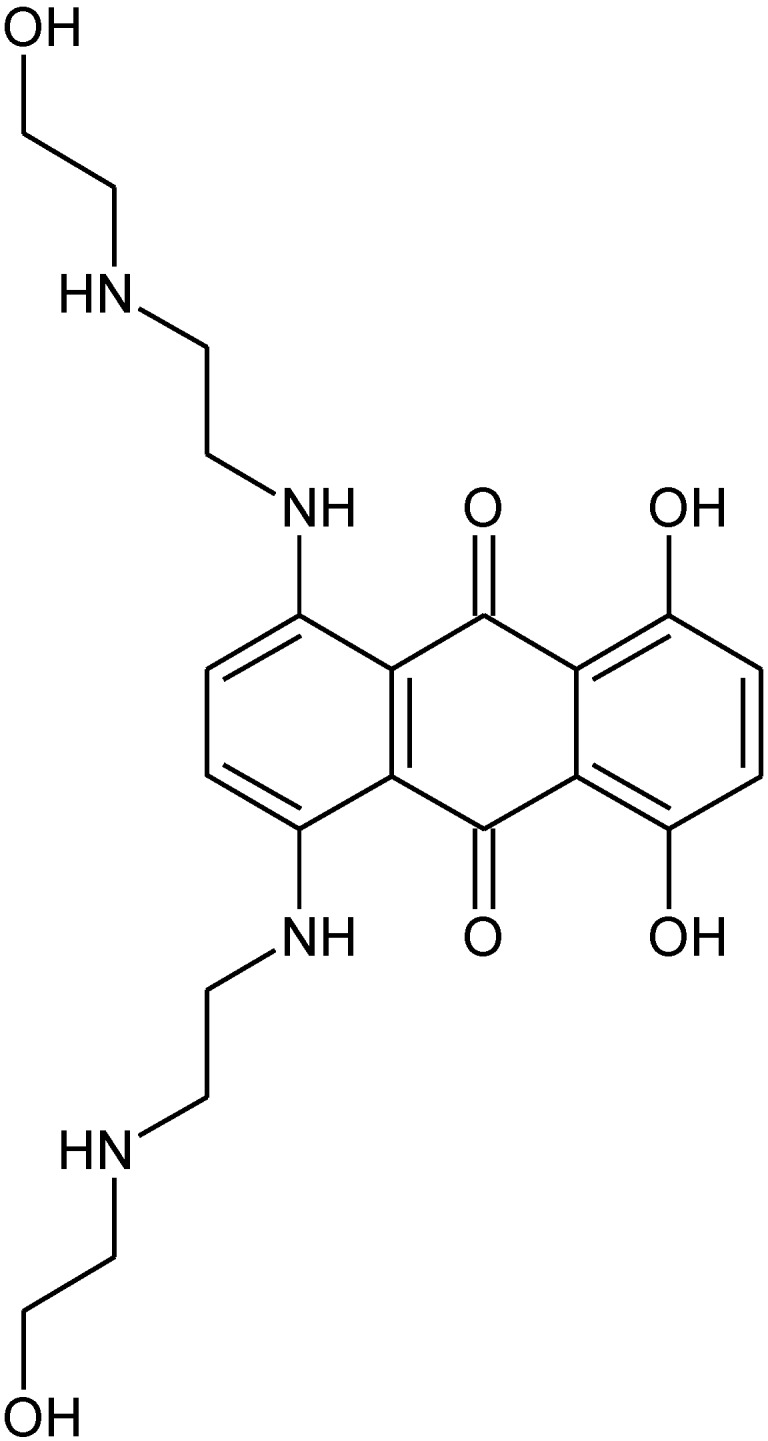
Mitoxantrone.

There are several possible consequences related to the difference in ability of different organotin polyethers to inhibit breast cancer cell lines based on the structure of the Lewis base moiety. First, other drugs incorporating known hormones may also demonstrate this preference for inhibiting the growth of non-estrogen sensitive breast cancer cells in comparison to estrogen sensitive breast cancer cells. Second, drugs containing related structures, specifically the O-phenylene or O-aromatic moiety, may also demonstrate a similar behavior. It is not known if this preference is operational when treating living breast cancer patients but it should be watched for, since a number of breast cancer treatments contain drugs that possess O-phenylene or O-aromatic moieties. These drugs include doxorubicin (Figure 4), epirubicin (Figure 5), mitoxantrone (Figure 6) and liposomal doxorubicin (the liposome of doxorubicin shown in Figure 4).

Most of the hormones employed in drug replacement therapy, such as DES and dienestrol, also contain this structural moiety. Our bodies contain an abundance of cations such as magnesium, calcium, tin, and iron that chelate with carbonyls and aromatic hydroxyls present in the aforementioned drugs currently employed in the treatment of breast cancer. Each of these cations contain vacant d orbitals that could then form a complex similar to that present in the Sn-O-phenylene moiety that may provide differentiation between the estrogen and non-estrogen sensitive breast cancer cells. Thus, it might be prudent to employ drugs that do not contain the Sn-O-phenylene moiety in treating MCF-7 related breast cancer or at least test for this differentiation.

The results are preliminary and must be viewed as such but do indicate that there exists a difference in the *in vitro* cell studies reported on here. Much more must be done before more definite conclusions should be reached.
